# Mutated p53 Promotes the Symmetric Self-Renewal of Cisplatin-Resistant Lung Cancer Stem-Like Cells and Inhibits the Recruitment of Macrophages

**DOI:** 10.1155/2019/7478538

**Published:** 2019-10-31

**Authors:** Yu Xu, Zhi Xu, Qi Li, Liang Guo, Yao Wang, Jianchun Zhou, Guansong Wang, Yuliang Liu

**Affiliations:** ^1^Department of Respiratory and Critical Care Medicine, Xinqiao Hospital, The Army Medical University, Chongqing, China; ^2^Department of Respiratory and Critical Care Medicine, The First Affiliated Hospital of Chongqing Medical University, Chongqing, China

## Abstract

It has been proposed that mutant p53 is correlated with the recurrence of lung cancer. Recently, a small population of cells with asymmetric or symmetric self-renewal potential has been identified in lung cancer, which was termed as cancer stem-like cells (CSCs) and was speculated to be the reason for cancer recurrence after chemotherapy. In this study, we used lung cancer cell lines with different TP53 backgrounds to elucidate the potential role of mutant p53 in regulating lung CSC self-renewal and on lung cancer recurrence. Cisplatin-resistant lung cancer cells with different TP53 backgrounds were generated *in vitro* by exposing A549, H460, and H661 lung cancer cell lines repeatedly to cisplatin. CD44^+^/CD90^+^ stem-like cells were identified in above cisplatin-resistant lung cancers (termed as cisplatin-resistant lung cancer stem-like cells, (Cr-LCSCs)) and stained with PKH26 dye which was used to define the self-renewal pattern. The proportion of symmetric divisions was significantly higher in Cr-LCSCs with mutant (mt) p53 compared with Cr-LCSCs with wild-type (wt) p53, and forced expression of mt p53 promoted the symmetric division of Cr-LCSCs. Furthermore, fewer macrophages accumulated in subcutaneously implanted xenografts consisting of mt p53 Cr-LCSCs compared with wt p53 Cr-LCSCs. These results indicated that mt p53 might accelerate the recurrence of lung cancer by regulating the self-renewal kinetics of Cr-LCSCs as well as the recruitment of macrophages.

## 1. Introduction

The lung is a barrier organ that is the first line of defense against various threats ranging from pathogens to carcinogens and is susceptible to cancer. Lung cancer is becoming the leading cause of cancer-related death in men and women [1]. Targeted drugs have been developed to treat lung cancer patients harboring EGFR mutations [2] or EML4-ALK amplification [3]. Immune checkpoint inhibitors (ICIs), namely, programmed death-1 (PD-1) antibodies [4], have been approved by the FDA as the first-line treatments. However, traditional cisplatin-based chemotherapy remains the first-line treatment for nonresectable lung cancer without actionable mutations or with PD-l tumor proportion scores (TPSs) that are less than 50%. A cisplatin-based chemotherapeutic strategy has been applied in patients with advanced IIIB or IV tumors and as an adjuvant therapy in earlier stages following surgery. However, the overall 5-year survival of NSCLC is under 40% [5], which is mainly attributed to the recurrence of lung cancer after chemotherapy.

It has been proposed that a small proportion of stem-like cells, termed as cancer-initiating cells (CIS) or cancer stem-like cells (CSCs), in tumors are responsible for the initiation, progression and, most importantly, the recurrence of cancer [6]. CSCs have been implicated in the recurrence of cancers by the ability to efflux chemotherapeutic drugs through the expression of several drug efflux and DNA repair proteins that are not eliminated after chemotherapy [7]. Besides, CSCs were divided symmetrically and asymmetrically similar to their normal counterparts, and the mode of propagation depends on the requirements of the stem cell pool reserve, tissue repair, and genetic background. Symmetrical division produces identical daughter cells that supply the stem cell pool that is required for rapid tissue repair, and asymmetric division produces one undifferentiated and one differentiated designated for reserving stem cell pool [8]. The regeneration of a tumor mass after chemotherapy may be influenced by the balance between symmetric and asymmetric cell divisions, and factors that determine this balance could result in the aberrant expansion of CSCs and recurrence of cancer.

Wild-type p53, which is translated by the tumor suppressor gene TP53, functions to prevent DNA damage. Mutant p53 leads to the dysfunction of wild-type p53. TP53 mutations have been identified in various cancer types, including lung cancer. It has been observed that mt p53 is related to a poor prognosis and the recurrence of lung cancer in resected and cisplatin-treated lung cancer [9, 10]. To understand the role of mt p53 in the recurrence of lung cancer, we investigated the involvement of mt p53 in regulating Cr-LCSC self-renewal and its interaction with the host immune system, especially macrophages of innate immunity. Thus, we aimed to accomplish the following goals: (1) isolate Cr-LCSCs from 3 lung cancer cell lines with different p53 backgrounds; (2) investigate the involvement of mt p53 in regulating the Cr-LCSC self-renewal pattern; (3) investigate the effect of mt p53 on the tumorigenicity of Cr-LCSCs; and (4) compare the density of macrophage infiltration in subcutaneously implanted wt and mt p53 Cr-LCSC xenografts. The results provided new insights and targets for the prevention of lung cancer recurrence after chemotherapy.

## 2. Materials and Methods

### 2.1. Cell Lines and Reagents

A549, H460, H661, and H1299 lung cancer cell lines were obtained from the American Type Culture Collection (ATCC) (Manassas, VA, USA) and cultured with RPMI-1640 medium (Gibco, Grand Island, NY, USA) supplemented with 10% fetal bovine serum (Hyclone, South Logan, UT, USA). Cisplatin, 4′,6-diamidino-2-phenylindole (DAPI), and a PKH-26 Red Fluorescent Cell Linker Kit were purchased from Sigma-Aldrich Chemical Company (St. Louis, MO, USA). FITC-conjugated CD44 antibody, APC-conjugated CD90 antibody, and corresponding isotype controls were purchased from BD Biosciences (San Jose, CA, USA). Anti-human CD44, CD90, and p53 primary antibodies were purchased from Abcam (Cambridge, MA, USA). CCR2 antibody was obtained from R&D (Minneapolis, MN, USA); CD68 antibody and Lipofectamine 2000 reagent were purchased from Invitrogen (Carlsbad, CA, USA). Secondary antibodies FITC-conjugated goat anti-rabbit IgG and Cy3-conjugated goat anti-mouse IgG were purchased from Jackson ImmunoResearch (West Grove, PA, USA). pCMV-Neo-Bam p53 R249S was a gift from Bert Vogelstein (Addgene plasmid # 16438), and pCMV-Neo-Bam p53 wt (wt p53) was a gift from Bert Vogelstein (Addgene plasmid # 16434).

### 2.2. Generation of Cisplatin-Resistant Lung Cancer Cell Lines

A549, H460, H661, and H1299 lung cancer cells were exposed to cisplatin according to the IC50 value for 72 h. The medium was removed, and the cells were cultured in normal medium until the cells fully recovered. Every 2-3 rounds of treatment, the IC50 concentrations were reassessed for each cell line. Cisplatin-resistant lung cancer cell lines were established when stable IC50 value was reached.

### 2.3. Isolation of Cr-LCSCs

CD44^+^/CD90^+^ cells were isolated from A549/Cr, H460/Cr, H661/Cr, and H1299/Cr cells using a MoFlo XDP flow cytometer stained with FITC-conjugated CD44 antibody and APC-conjugated CD90 antibody. Cell debris and dead cells doublets were gated based on cell size and complexity.

### 2.4. Immunofluorescence Staining

A549/Cr, H460/Cr, and H661/Cr cells were cultured on coverslip, fixed in 4% paraformaldehyde, and then labeled with anti-human CD44 and CD90 at 4°C overnight. The samples were incubated with FITC-conjugated goat anti-rabbit IgG and Cy3-conjugated goat anti-mouse IgG (1 : 200 dilution) for 1 h at 37°C. To stain macrophages in the tumors, frozen tissues were cut into 10 *μ*m thick sections, fixed in cold acetone for 10 min, and blocked with 10% normal goat serum. The tumor sections were incubated with a primary anti-CD68 antibody (1 : 200 dilution) overnight in a humid chamber at 4°C, then incubated with a FITC-conjugated goat anti-rabbit IgG secondary antibody for 1 h at 37°. Nuclei were counter-stained with DAPI. Images were acquired by confocal microscopy.

### 2.5. Immunohistochemical (IHC) Analysis

The formalin-fixed, paraffin-embedded xenograft tissues were cut into 8 *μ*m thick sections. Slides were dewaxed, put in microwave for antigen retrieval at 95 for 30 min, and then incubated with a CCR2 antibody (1 : 200 dilution), and color was developed by the DAB method. Microscopic images were acquired by phase contrast microscopy (BX41TF, OLYMPUS, Tokyo, Japan). CellSens Standard 1.13 (OLYMPUS) software was used to capture the images.

### 2.6. Transfection of wt p53 and mt p53 in H1299/Cr Cells

4 *μ*g of DNA of the control pCMV-Neo-Bam vector, pCMV-Neo-Bam p53 R249S vectors, and pCMV-Neo-Bam p53 wt vectors was mixed with Lipofectamine 2000 reagent in Opti-MEM Medium, incubated for 5 min at room temperature, and then added to H1299/Cr cells at 70% confluent and incubated for 48 h. 300 *μ*g/mL of G418 was used to select stably transfected clones.

### 2.7. PKH-26 Staining

PKH-26 is a cell membrane fluorescent dye and is divided to daughter cells. The distribution of PKH-26 in the daughter cells was tracked, and a mathematic model provided by Cicalese et al. was used to define the division patterns [11]. PKH-26 dye was diluted as indicated and incubated with PBS-washed cells for 5 min at room temperature. The staining reaction was ceased by adding 2 ml serum. The cells were washed and centrifuged to remove unbound excess dye, resuspended in serum-free DMEM medium supplemented with 20 ng/ml EGF, 20 ng/ml bFGF, and 4 *μ*g/ml insulin. Single cells were obtained by serial dilutions in a 96-well plate, labeled, and observed under fluorescence microscopy. The images were acquired by confocal microscopy.

### 2.8. Sphere Formation Assay

To assess the spheroid formation ability, Cr-LCSCs were cultured in serum-free DMEM medium supplemented with 20 ng/ml EGF, 20 ng/ml bFGF, and 4 *μ*g/ml insulin for 3 weeks. The sphere formation ability was assessed by the time of appearance and the number and size of sphere bodies and plotted as histograms.

### 2.9. Tumor Xenografts in Nude Mice

BALB/c nude mice were used to establish tumor xenografts by injection of a 200 *μ*l cell suspension of 1 × 10^5^ H1299/Cr-mt-p53 and H1299/Cr-wt-p53 cells subcutaneously. Tumor volumes (mm^3^) were calculated every week by the following formula: (major axis)∗(minor axis)^2^/2. At 4 weeks, the mice were sacrificed, and the tumors were removed and measured at necropsy.

### 2.10. Statistical Analysis

The results were presented as the mean ± standard deviation (SD). Differences were analyzed with two-tailed unpaired Student's *t*-test between 2 groups or one-way ANOVA between 3 groups. *p* < 0.05 was defined statistically significant. The data were analyzed using SPSS v16.0 software (SPSS Inc., Chicago, IL, USA), and graphs were performed using GraphPad Prism software (La Jolla, CA, USA).

## 3. Results

### 3.1. Isolation of Cr-LCSCs from Cisplatin-Resistant Nonsmall Cell Lung Cancer Cell Lines

To recapitulate the recurrence of LCSCs following chemotherapy, A549 (wt P53), H460 (wt P53), and H661 (mt P53) human lung cancer cell lines were treated with cisplatin at concentrations ranging from 0.1 *μ*M to 100 *μ*M to determine the IC50 for the initial treatment, as previously reported [12]. The cells were then treated with cisplatin according to the IC50 for 72 h, and the remaining cells were cultured in RPMI-1640 complete medium until full recovery (Fig. [Supplementary-material supplementary-material-1]). After approximately 20 cell passages with the treatment, the cells began to show stable IC50 values, and cells after 20 passages were defined as cisplatin-resistant (Cr) cells and used for subsequent investigations (Fig. [Supplementary-material supplementary-material-1]) [12].

**Figure 1:**
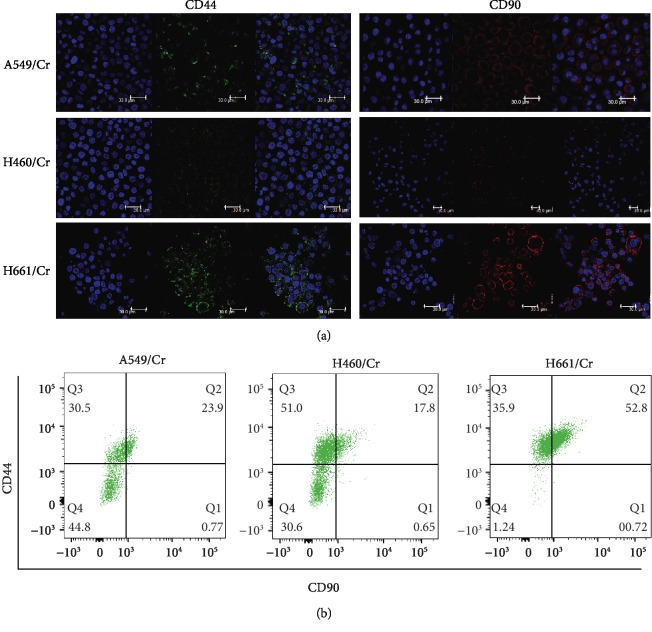
Increased LCSC markers in cisplatin-resistant lung cancer cell lines. (a) Immunostaining of the LCSC markers CD44 and CD90 in A549/Cr, H460/Cr, and H661/Cr cells. Scale bar = 30 *μ*m. (b). FACS analysis of CD44^+^/CD90^+^ LCSCs in A549/Cr, H460/Cr, and H661/Cr cells.

CD44 [13] and CD90 [14] are LCSC surface markers. We analyzed the expression of CD44 and CD90 in A549/Cr, H460/Cr, and H661/Cr cells using an immunofluorescence assay. CD44 and CD90 were detected in A549/Cr, H460/Cr, and H661/Cr cells (Figure 1(a)). Since CD44^+^/CD90^+^ were defined as LCSCs previously [90], we detected the proportion of CD44^+^/CD90^+^ cells in A549/Cr, H460/Cr, and H661/Cr cells. CD44^+^/CD90^+^ cells were identified in A549/Cr, H460/Cr, and H661/Cr cells, indicating LCSCs were enriched with cisplatin treatment in vitro (Figure 1(b)). The above double-positive cell population was designated as cisplatin-enriched LCSCs (Cr-LCSCs). Interestingly, we found the proportion of Cr-LCSCs in mt p53 H661/Cr was significantly higher compared with those in wt p53 A549/Cr and H460/Cr (Figure 1(b) and Fig. [Supplementary-material supplementary-material-1]).

### 3.2. A549/Cr-LCSCs, H460/Cr-LCSCs, and H661/Cr-LCSCs Exhibited Discrepant Self-Renewal Properties and Propagated Differently

**Figure 2:**
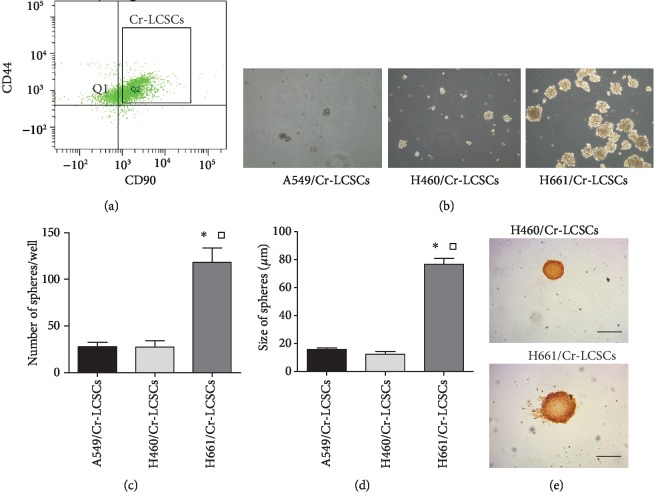
The isolation and continuous culture of Cr-LCSCs obtained from A549/Cr, H460/Cr, and H661/Cr cells. (a) Representative FACS plots demonstrating the isolation of CD44^+^/CD90^+^ LCSCs from A549/Cr, H460/Cr, and H661/Cr cells. (b) Bright field images of spheres generated from clonal density cultures of A549/Cr-LCSCs, H460/Cr-LCSCs, and H661/Cr-LCSCs collected 21 days after the start of the suspension cultures (100x magnification). (c) The number of A549/Cr-LCSC, H460/Cr-LCSC, and H661/Cr-LCSC spheres in 100 *μ*l samples. The data are presented as the mean ± SD for triplicate counts. ^∗^*p* < 0.05 compared with A549/Cr-LCSCs and ^□^*p* < 0.05 compared with H460/Cr-LCSCs. (d) The sizes (*μ*m) of the A549/Cr-LCSC, H460/Cr-LCSC, and H661/Cr-LCSC spheres. ^∗^*p* < 0.05 compared with A549/Cr-LCSCs and ^□^*p* < 0.05 compared with H460/Cr-LCSCs. (e) H460/Cr-LCSCs and H661/Cr-LCSCs were labeled with BrdU and identified with an anti-BrdU antibody. Scale bar = 50 *μ*m.

To further study the intrinsic difference between Cr-LCSCs from A549/Cr, H460/Cr, and H661/Cr cells, which might be regulated by p53 status, we first isolated CD44^+^/CD90^+^ subpopulations using flow cytometry (Figure 2(a) and Fig. [Supplementary-material supplementary-material-1]). A total of 1 × 10^4^ sorted A549/Cr-LCSCs, H460/Cr-LCSCs, and H661/Cr-LCSCs were cultured in 24-well nonadherent plates in serum-free medium supplemented with EGF, bFGF, and insulin. Mutant P53 H661/Cr-LCSCs formed spheres in 3 days, and wild-type P53 A549/Cr-LCSC and H460/Cr-LCSC spheres appeared after 10 days of culture (Figure 2(b)), indicating that Cr-LCSCs exhibited self-renewal characteristics in vitro. After 3 weeks, we found that the number of H661/Cr-LCSC spheres was significantly greater than that of A549/Cr-LCSC and H460/Cr-LCSC spheres that were counted under microscopy (Figure 2(c)), and the measured size of the H661/Cr-LCSC spheres was larger than that of A549/Cr-LCSC and H460/Cr-LCSC spheres (Figure 2(d)). After staining the spheres with BrdU, no significant differences were observed in the proliferation of the wt p53 H460/Cr-LCSC and mt p53 H661/Cr-LCSC spheres (Figure 2(e)). The results showed that mt p53 influences Cr-LCSC propagation through mechanisms rather than manipulating proliferation.

**Figure 3:**
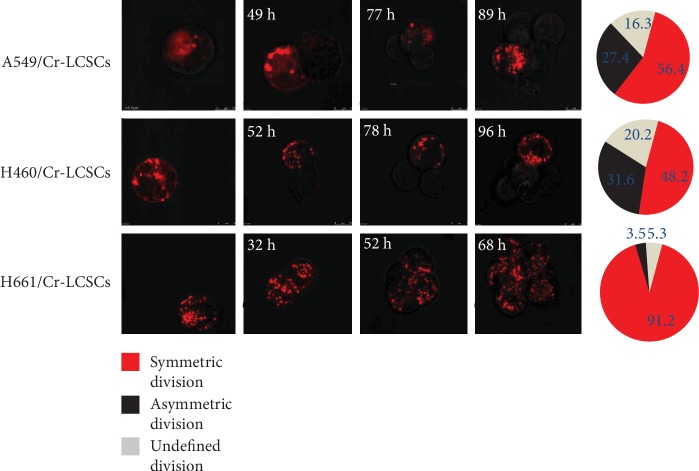
Fluorescence microscopy for the analysis of A549/Cr-LCSC, H460/Cr-LCSC, and H661/Cr-LCSC divisions following cell seeding. The accompanying pie charts indicate the relative frequencies of asymmetric and symmetric cell divisions. Undefined divisions are division patterns that could not be determined due to the loss of images during culture or an indistinct cell number following division.

The number of stem cells can be regulated by the balance between symmetric and asymmetric mitotic cell divisions. To further investigate the role of mt p53 on the division pattern of Cr-LCSCs, the above-mentioned cells were investigated using PKH-26 cell staining, as described previously [11, 12]. In this study, we found that the pattern of the cell division of the A549/Cr-LCSCs was 56.3% symmetric, 27.4% asymmetric, and 16.3% undefined (could not be classified by the PKH-26 dye using a mathematical model provided by Cicalese et al.). The cell division of the H460/Cr-LCSCs was 48.2% symmetric, 31.6% asymmetric, and 20.2% undefined. The pattern of the cell division of the H661/Cr-LCSCs was 91.2% symmetric, 3.5% asymmetric, and 5.3% undefined (Figure 3). These results were consistent with a previous study showing that drug-resistant NSCLCs are mainly divided symmetrically [12]. Furthermore, this study indicated that mt p53H661/Cr-LCSCs nearly exclusively preferred symmetric division compared with wt p53 A549/Cr-LCSCs and H460/Cr-LCSCs, providing a reasonable explanation for the slower self-renewal rate of A549/Cr-LCSCs and H460/Cr-LCSCs.

### 3.3. mt p53 Promoted the Symmetric Self-Renewal of Cr-LCSCs

**Figure 4:**
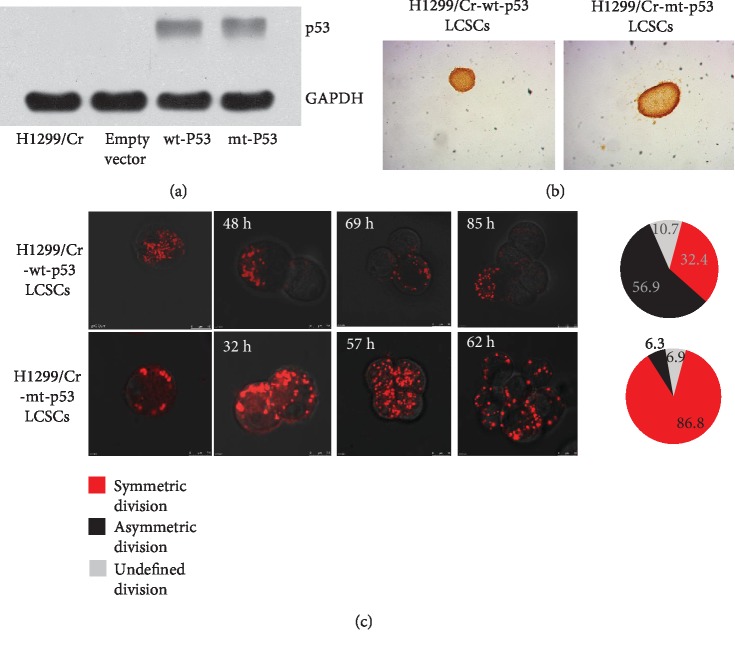
mt p53 promotes the symmetric cell division of cisplatin-resistant stem cell-like NSCLC cells. (a) Forced expression of wt p53 and mt p53 in p53 null H1299/CisR cells. Western blot analysis of lysates from H1299/Cr, H1299/Cr-wt-p53, and H1299/Cr-mt-p53 cells using an anti-p53 antibody. GAPDH was used as an internal control. (b, c) Fluorescence microscopy for the analysis of the division of CD44^+^/CD90^+^ LCSCs isolated from H1299/Cr-wt-p53 and H1299/Cr-mt-p53 cells following cell seeding.

To further investigate the role of mt p53 on the self-renewal of Cr-LCSCs, a p53 null H1299 lung cancer cell line was treated with cisplatin to generate H1299/Cr. We transfected H1299/Cr with wt p53 and mt p53 plasmids to obtain stably transfected cell lines, named H1299/Cr-wt-p53 and H1299/Cr-mt-p53, respectively. The degree of wt p53 and mt p53 overexpression in the stable clones was verified by western blot (Figure 4(a)). Furthermore, we isolated CD44^+^/CD90^+^ cells from H1299/Cr-wt-p53 and H1299/Cr-mt-p53 cells and cultured in serum-free medium. We stained Cr-LCSC spheres with BrdU to exclude the difference in proliferation; as expected, overexpression of wt p53 or mt p53 had no effect on the proliferation of Cr-LCSC spheres (Figure 4(b)). Then we studied the effect of overexpression of wt p53 or mt p53 on Cr-LCSC division with the PKH-26 staining. The division of LCSCs from H1299/Cr-wt-p53 cells was 32.4% symmetric, 56.9% asymmetric, and 10.7% undefined, and the division of LCSCs from H1299/Cr-mt-p53 cells was 86.8% symmetric, 6.3% asymmetric, and 6.9% undefined (Figure 4(c)). These results further demonstrated that mt p53 is capable of manipulating Cr-LCSC division towards a symmetric pattern.

### 3.4. mt p53 Cr-LCSCs Showed Increased Tumorigenicity in a Subcutaneous Xenograft Model and Suppressed the Accumulation of Macrophages

**Figure 5:**
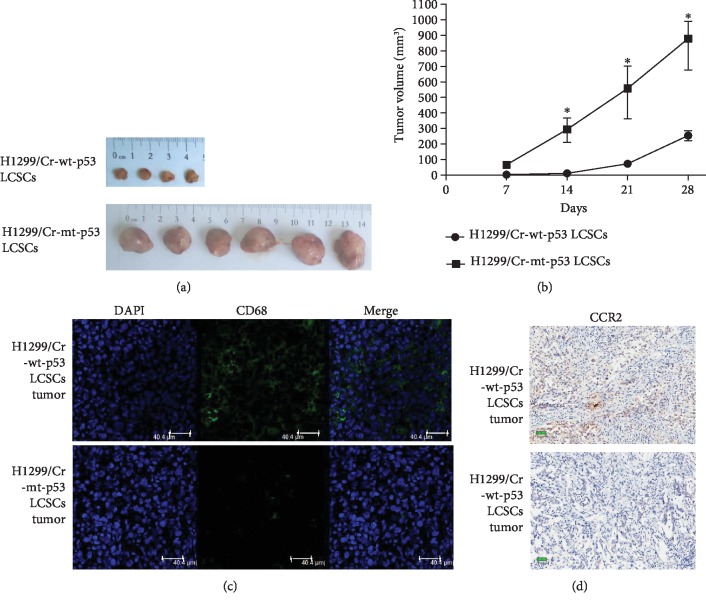
mt P53 promoted Cr-LCSC xenograft progression in nude mice and prevented the accumulation of macrophages at the tumor site. (a) The appearance of subcutaneous tumors in H1299/Cr-mt-p53 LCSC and H1299/Cr-wt-p53 LCSC xenografts. Four out of 6 mice formed subcutaneous tumors after the injection of H1299/Cr-wt-p53 LCSCs, while 6 out of 6 mice formed subcutaneous tumors after the injection of H1299/Cr-mt-p53 LCSCs. (b) Tumor volume growth curves of tumors developed in the xenograft models injected with H1299/Cr-mt-p53 and H1299/Cr-wt-p53 LCSCs. (c) Immunofluorescence staining showing the accumulation of CD68^+^ macrophages in H1299/Cr-wt-p53 LCSC xenografts and the scant accumulation of macrophages in H1299/Cr-mt-p53 LCSC xenografts. Scale bar = 40 *μ*m. (d) IHC staining showed decreased CCR2 expression levels in H1299/Cr-mt-p53 LCSC xenografts. Scale bar = 50 *μ*m.

We examined the in vivo tumorigenicity of mt p53 Cr-LCSCs and wt p53 Cr-LCSCs using subcutaneous injections of CD44^+^/CD90^+^ cells isolated from H1299/Cr-wt-p53 and H1299/Cr-mt-p53 cells. The growth of subcutaneous tumors was monitored and measured every week until the fourth week. The growth rate of tumors derived from H1299/Cr-mt-p53 LCSCs was markedly accelerated compared with H1299/Cr-wt-p53 LCSCs (Figure 5(a)). At the end of four weeks, 2 out of 6 mice injected with H1299/Cr-wt-p53 cells fail to develop tumors while all mice injected with H1299/Cr-mt-p53 cells developed. The volume of tumors derived from H1299/Cr-mt-p53 LCSCs was markedly larger compared with that from H1299/Cr-wt-p53 LCSCs (Figure 5(b)).

To further explore the increased tumorigenicity of mt p53 Cr-LCSCs in terms of the host immune defense, we focused on the macrophages of the host immune system because athymic nude mice are not suitable for the investigation of acquired immunity. Xenografts from H1299/Cr-wt-p53 and H1299/Cr-mt-p53 LCSCs were stained with a CD68 macrophage marker, and a decrease in CD68^+^ macrophages was observed in H1299/Cr-mt-p53 xenografts compared with H1299/Cr-wt-p53 xenografts (Figure 5(c)). CCR2 has been proposed to accumulate macrophages at the tumor site, and we investigated the expression of CCR2 in H1299/Cr-wt-p53 and H1299/Cr-mt-p53 LCSC xenografts. We found that CCR2 expression was downregulated in H1299/Cr-mt-p53 LCSC xenografts compared with that in H1299/Cr-wt-p53 LCSC xenografts (Figure 5(d)).

## 4. Discussion

Platinum-based doublet chemotherapy is the standard first-line therapy for patients with NSCLC without actionable “driver genes” or a PD-L1 TPS < 50%. 20-40% of patents tend to relapse within 6 months, although with initial response to platinum-based doublet chemotherapy in lung cancer [15]. Many previous studies have proposed that this may be due to the existence of a chemotherapy-resistant phenotype with stem cell-like traits during chemotherapy treatment. Evidence has indicated that LCSCs are responsible for lung cancer recurrence because cytotoxic reagents eliminate the bulk of differentiated tumor cells, while LCSCs survive and continue to proliferate [12]. Theoretically, similar to its normal adult stem cell counterpart, LCSCs are predicted to remain quiescent and should have a slower growth rate than differentiated tumor cells. However, evidence has shown that the environment, signaling pathways, and epigenetic changes maintain the balance between the differentiation and quiescence of LCSCs. We proposed that LCSCs might be activated during chemotherapeutic treatment and must quickly respond to expand and regenerate a damaged tumor mass. Using the method reported by Barr [16], we enriched drug-resistant lung cancer cells with stem cell properties after ~20 passages using cisplatin in three lung cancer cell lines that were designated as A549/Cr, H460/Cr, and H661/Cr cells. CD44 and CD90 have been reported to be markers of LCSCs, and CD44^+^/CD90^+^ LCSCs were isolated from A549/Cr, H460/Cr, and H661/Cr cells. In this study, we investigated the self-renewal, tumorigenicity, and immunogenicity of Cr-LCSCs and the regulatory factors.

During tissue repair, symmetric division is adopted by stem cells to generate more progeny for a quick response to expand and replace damaged cells. The tumor mass can be considered an abnormal organ in which CSCs might respond to damage, such as damage from chemotherapy and radiotherapy, that disrupts the normal balance between asymmetric division and symmetric division. Whether Cr-LCSCs adopt symmetric division or symmetric division under chemotherapy is unknown. In this study, to determine the self-renewal mode of isolated Cr-LCSCs, we used PKH-26 to label progeny of Cr-LCSCs. For symmetric division, PKH-26 is distributed equally to two daughter cells that are destined to have the same fate of either differentiation or maintained stemness. Asymmetric division results in the retention of the PKH-26 dye in daughter stem cells, which are relatively more quiescent compared with differentiated progeny. The results showed that the majority of Cr-LCSCs isolated from lung cancer cell lines underwent symmetric division, indicating that exposure to chemotherapy in vitro promotes the symmetric self-renewal of LCSCs. The in vivo impact of chemotherapy on CSC self-renewal has also been reported in patients with breast cancer who received neoadjuvant chemotherapy because the self-renewal potential to form mammospheres was enhanced after neoadjuvant chemotherapy [17].

TP53 is a tumor suppressor gene that determines the cell fate of stressed or damaged cells by inducing reversible cell cycle arrest, DNA repair, or apoptosis [18]. TP53 gene mutations are frequent in NSCLC, and the mutations result in incorrect protein synthesis or alterations in the DNA-binding domain that impairs the tumor suppressor function of p53 [19]. Clinical data describing mutant p53 determining the prognosis of patients with NSCLC have been widely reported [20, 21]. In stage I NSCLC, the risk of death increased in patients with tumor p53 mutations compared with tumor p53 wt [22]. p53 null mutation also leads to a poor outcome in patients with early-stage NSCLCs [23]. The tendency of a better prognosis in NSCLCs patients with wt p53 was presumed to be associated with the tumor suppressor activities of wt p53, while tumors with mt p53 escaped DNA damage-dependent cell senescence and apoptosis induced by chemotherapy or radiotherapy. Recently, p53 was reported to regulate the self-renewal mode of stem cells [11]. The effect of mt p53 on the self-renewal of Cr-LCSCs has not been investigated. In this study, we reported that mt p53 promotes the symmetric cell division of Cr-LCSCs, leading to the possibility of the accumulation of a stem cell pool in mt p53 NSCLC tumor masses following cisplatin treatment.

To further elucidate the possible role of mt p53 on the recurrence of lung cancer, we compared the tumorigenicity of mt p53 Cr-LCSCs and that of wt p53 Cr-LCSCs in vivo. The mt p53 Cr-LCSCs are more tumorigenic compared with wt P53 Cr-LCSCs and have an increased tumor growth rate and end-point tumor mass. Mononuclear phagocytes are present in the lung from the earliest stages of lung development until death and are the most important innate immune system components in the lung that defend against cancer. The infiltration of leukocyte (including macrophages) in tumor was thought to be the result of an immune reaction to the tumor itself, specifically, the first innate immunity and later specific immunity to recognize tumor-associated antigens [24]. CD68^+^ macrophage infiltration in esophageal cancer is associated with better prognosis [25]. However, tumor-associated macrophages are recently thought to promote tumor initiation, growth, and development [26]. The role of tumor-associated macrophage in cancer is still controversial [27]. In this study, we explored the accumulation of macrophages in mt p53 Cr-LCSC and wt p53 Cr-LCSC xenografts. We found that mt p53 inhibited the accumulation of macrophages in xenografts with decreased CCR2 expression levels. It has been reported that p53 is a suppressor of inflammatory response in mice by repressed expression of inflammatory chemokine receptor CCR2 [28]. Whether mt p53 has a stronger repression effect on CCR2 needs further investigation. CCR2 is the most important chemokine receptor in the recruitment of macrophages in cancer; genetic deletion of CCR2 results in less infiltration of macrophages in mouse xenografts [29]. Our results showed that mt p53 inhibited CCR2 and resulted in decreased macrophages in xenografts and revealed a potential cross-talk between cancer cells and macrophage recruitment modulated by p53 somatic mutation in cancer.

Overall, this study suggested the possibility that current anticancer chemotherapy fail to eradicate resistant LCSC clones with symmetric cell division pattern may be associated with poor responses and lung cancer recurrence. mt p53 activation further increased Cr-LCSC expansion by promoting symmetric self-renewal division and inhibiting the accumulation of macrophages, resulting in increased tumorigenicity.

## Data Availability

Supplementary data was available.

## Supplementary Materials

Supplementary Materials Fig. S1: the morphology of A549/CisR, H460/CisR, and H661/CisR cells. Fig. S2: the IC50 for cisplatin treatment in vitro. Fig. S3: the proportion of CD44^+^/CD90^+^ cells in A549/CisR, H460/CisR, and H661/CisR cells. Fig. S4: FACS gating strategy of Cr-LCSCs. Fig. S5: flow cytometry analysis of CD44^+^/CD90^+^ cells in 3rd-generation SB (sphere body) of A549/CisR (CD44^+^/CD90^+^), H460/CisR (CD44^+^/CD90^+^), H661/CisR (CD44^+^/CD90^+^), and H1299/CisR (CD44^+^/CD90^+^) cells. Data are presented as the mean ± SD for triplicate counts. ^∗^*p* < 0.05, compared with A549/CisR (CD44^+^/CD90^+^); ^□^*p* < 0.05, compared with H460/CisR (CD44^+^/CD90^+^); ^△^compared with H661/CisR (CD44^+^/CD90^+^); ^◇^compared with H1299/CisR (CD44^+^/CD90^+^).
