# Supplementary material for: Mutated p53 Promotes the Symmetric Self-Renewal of Cisplatin-Resistant Lung Cancer Stem-Like Cells and Inhibits the Recruitment of Macrophages
Source: J Immunol Res. 2019 Oct 31;2019:7478538. doi: 10.1155/2019/7478538 (PMC6875234; doi:10.1155/2019/7478538)
Supplement: Supplementary Materials — Fig. S1: the morphology of A549/CisR, H460/CisR, and H661/CisR cells. Fig. S2: the IC50 for cisplatin treatment in vitro. Fig. S3: the proportion of CD44+/CD90+ cells in A549/CisR, H460/CisR, and H661/CisR cells. Fig. S4: FACS gating strategy of Cr-LCSCs. Fig. S5: flow cytometry analysis of CD44+/CD90+ cells in 3rd-generation SB (sphere body) of A549/CisR (CD44+/CD90+), H460/CisR (CD44+/CD90+), H661/CisR (CD44+/CD90+), and H1299/CisR (CD44+/CD90+) cells. Data are presented as the mean ± SD for triplicate counts. ∗p < 0.05, compared with A549/CisR (CD44+/CD90+); □p < 0.05, compared with H460/CisR (CD44+/CD90+); △compared with H661/CisR (CD44+/CD90+); ◇compared with H1299/CisR (CD44+/CD90+). [file 7478538.f1.doc]

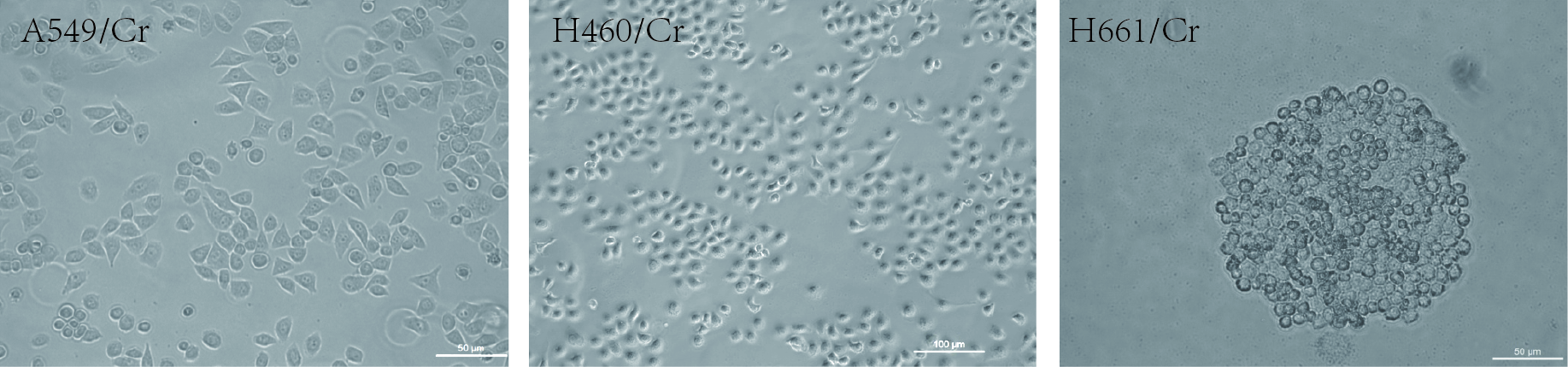


Fig. S1. Morphology of A549/CisR, H460/CisR, H661/CisR cells at 100x magnification.Scale bar = 50 μm


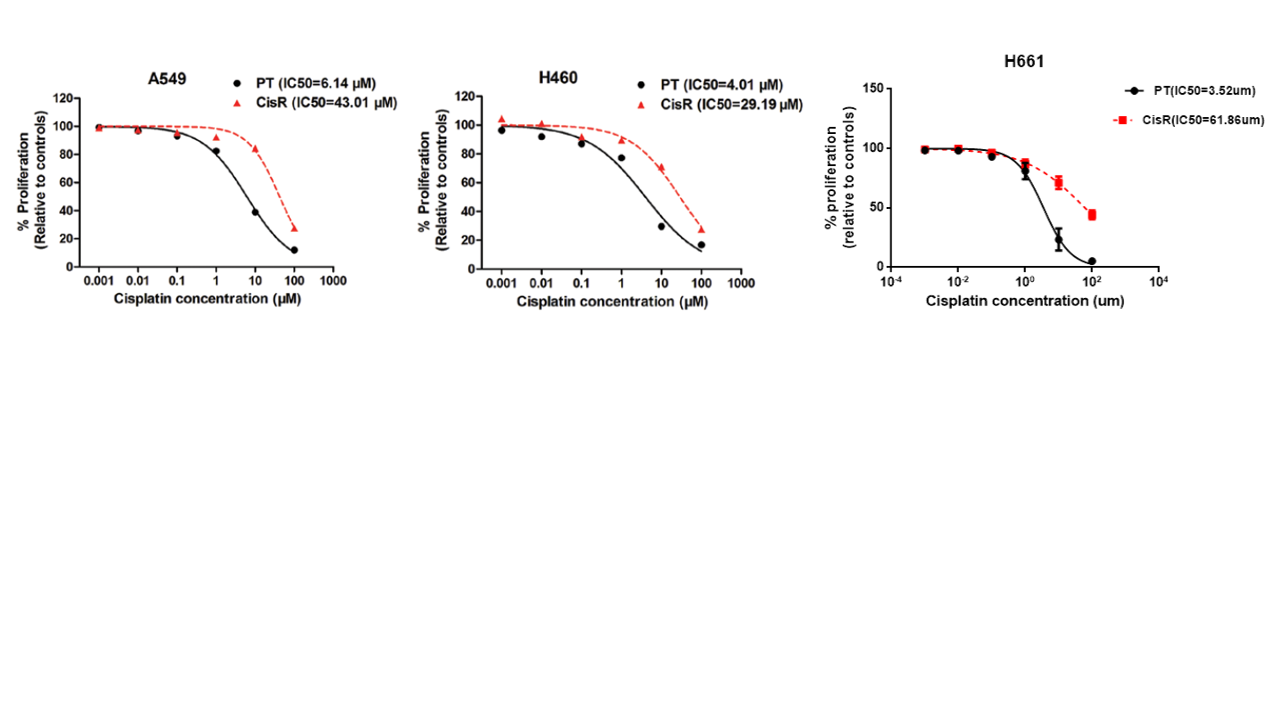


Fig. S2. The IC50 concentrations detected in A549, H460, H661 Cisplatin-resistant cell lines and the parental cell lines.


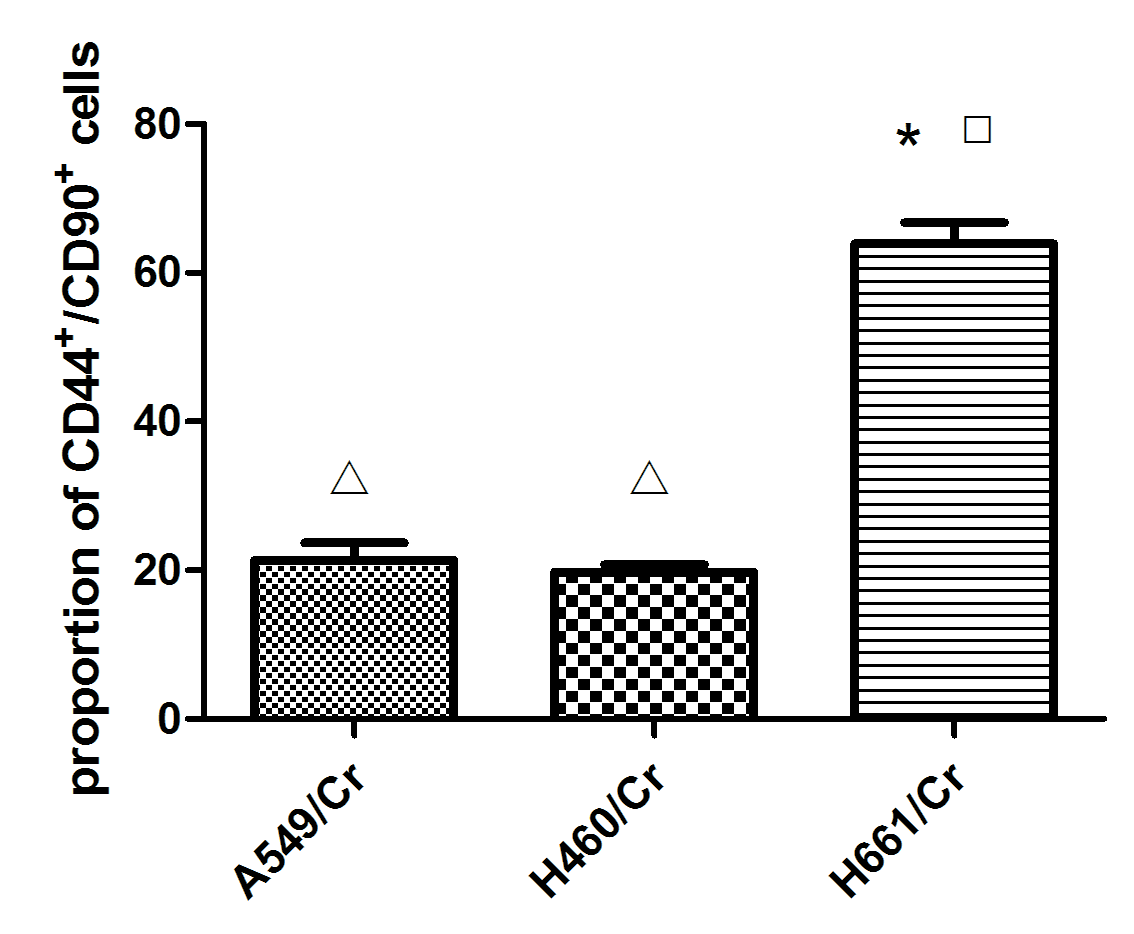


Fig. S3. The proportion of CD44^+^/CD90^+^ cells in A549/CisR, H460/CisR, H661/CisR detected by flow cytometry. Data arepresented as the mean ± SD for triplicate counts. *, p<0.05 compared with A549/CisR , □, p<0.05 compared with H460/CisR, △, compared with H661/CisR.


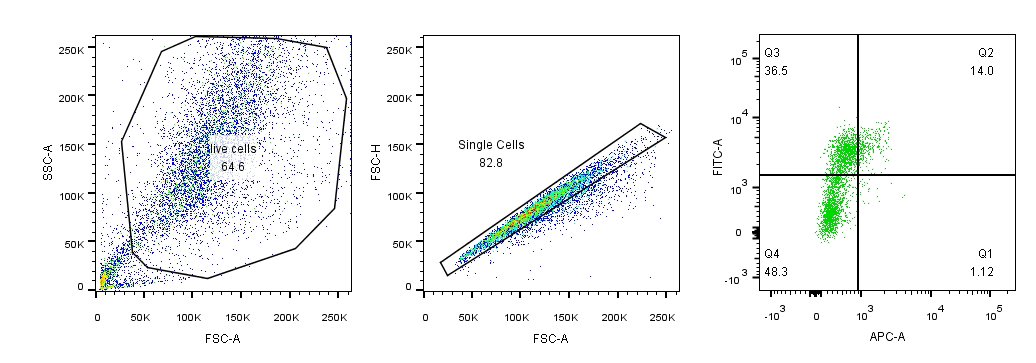


Fig. S4. FACs gating strategy of Cr-LCSCs. A549/CisR, H460/CisR, H661/CisR cells were trypsinized into single cells, stained with FITC-CD44 and APC-CD90 antibody and gated using flow cytometer. Appropriate compensation between fluorochrome has been carried out, After the exclusion of debris and doublets,Cr-LCSCs were identified and sorted by CD44 and CD90 double staining (Q2).


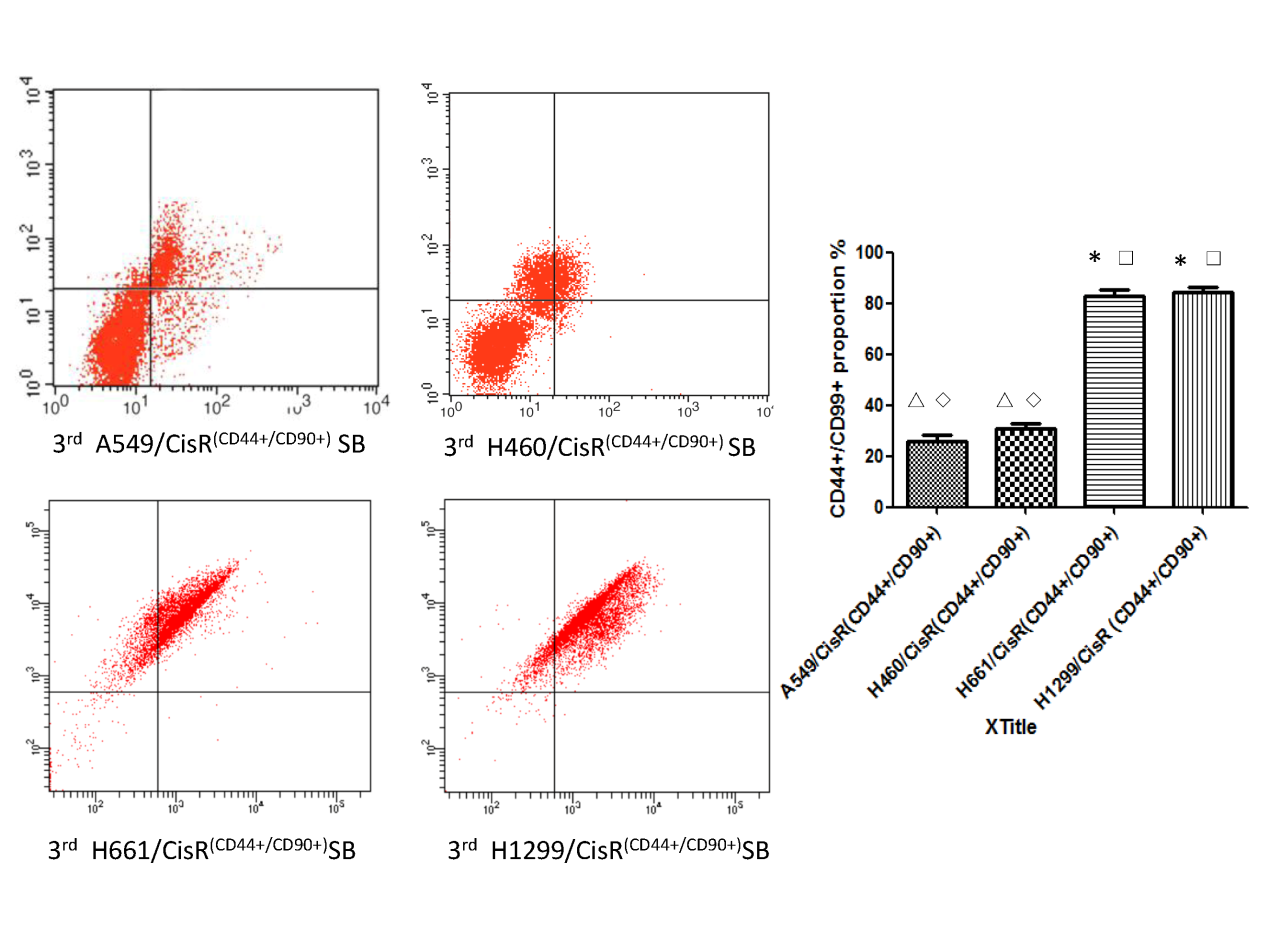


Fig. S5. Flow cytometry analysis of CD44+/CD90+ cells in 3rd generation SB (sphere body) of A549/CisR^(CD44+/CD90+)^, H460/CisR^(CD44+/CD90+)^, H661/CisR ^(CD44+/CD90+)^ and H1299/CisR ^(CD44+/CD90+)^ cells. Data are presented as the mean ± SD for triplicate counts. *, p<0.05 compared with A549/CisR^(CD44+/CD90+)^ , □ , p<0.05 compared with H460/CisR^(CD44+/CD90+)^, △, compared with H661/CisR^(CD44+/CD90+)^, ◇, compared with H1299/CisR^(CD44+/CD90+)^.
